# Mechanistic intersections of empagliflozin and nebivolol in aging-associated redox and inflammatory pathways

**DOI:** 10.3389/fphar.2026.1842207

**Published:** 2026-07-16

**Authors:** Mohammed Akram Al-Mahdawi, Hayder Adnan Fawzi, Nurul'Izzah Ibrahim, Norliza Muhammad

**Affiliations:** 1 Department of Pharmacology, Faculty of Medicine, Universiti Kebangsaan Malaysia (UKM), Kuala Lumpur, Malaysia; 2 Department of Clinical Pharmacy, College of Pharmacy, Al-Mustafa University, Baghdad, Iraq

**Keywords:** aging, empagliflozin, inflammaging, mitochondrial dysfunction, nebivolol, oxidative stress

## Abstract

Aging is characterized by interconnected disturbances in oxidative stress, chronic inflammation, mitochondrial dysfunction, and nutrient-sensing pathways that collectively contribute to progressive metabolic and vascular decline. Among the central molecular mechanisms implicated in these processes are dysregulation of the AMPK–mTOR–SIRT1 axis, persistent NF-κB activation, impaired Nrf2-mediated antioxidant defense, and mitochondrial redox imbalance. Pharmacological strategies capable of modulating multiple components of this network have therefore gained increasing interest in aging-related research. Empagliflozin, a sodium–glucose cotransporter-2 (SGLT2) inhibitor, has demonstrated pleiotropic metabolic effects extending beyond glycemic control, including modulation of AMPK signaling, attenuation of oxidative stress, and suppression of inflammatory activation. Nebivolol, a third-generation β_1_-selective adrenergic blocker with nitric oxide–mediated vasodilatory properties, has been associated with endothelial protection, vascular redox regulation, and anti-inflammatory activity. Although both agents influence pathways relevant to aging biology, current evidence is derived predominantly from experimental models of cardiovascular or metabolic disease rather than from physiological aging studies. Moreover, direct evidence evaluating their combined effects in aging remains unavailable. This review critically examines the mechanistic roles of empagliflozin and nebivolol within the context of aging-associated metabolic and vascular dysfunction. Particular emphasis is placed on their reported interactions with AMPK–mTOR–SIRT1 signaling, Nrf2-dependent antioxidant responses, NF-κB–mediated inflammation, mitochondrial function, and endothelial homeostasis. In addition, the review discusses current limitations in the evidence base, including the predominance of reductionist experimental approaches, limited translational validation, and the absence of direct combinational investigations. Comparative consideration is also given to established aging-relevant candidates, including metformin, rapamycin, resveratrol, GLP-1 receptor agonists, NAD^+^ modulators, and senolytic strategies, in order to contextualize the potential relevance and limitations of metabolic–vascular pathway modulation in aging research. Rather than proposing definitive evidence of aging-relevant efficacy, the available literature supports a biologically plausible framework in which modulation of metabolic and vascular pathways may influence interconnected mechanisms underlying aging-related decline. Further integrative experimental and translational studies are required to determine whether coordinated targeting of these pathways may provide meaningful therapeutic benefit in aging and age-associated disorders.

## 1 Introduction

Aging is a progressive and multifactorial biological process characterized by the gradual loss of cellular and systemic homeostasis (Maldonado et al., 2023), resulting in functional decline across organ systems. At the molecular level, one of the most consistent and reproducible features of aging is the convergence of oxidative stress, chronic low-grade inflammation, and mitochondrial dysfunction (Arab et al., 2022). These processes are not independent events but form an interconnected pathogenic network that drives metabolic and vascular deterioration over time.

Excessive production of reactive oxygen species (ROS), coupled with diminished antioxidant defenses, disrupts the redox equilibrium and activates redox-sensitive transcription factors, such as nuclear factor-κB (NF-κB) (Hong et al., 2024). Sustained NF-κB activation promotes transcription of pro-inflammatory mediators (Salminen et al., 2012), including tumor necrosis factor-α (TNF-α) and interleukin-6 (IL-6), thereby establishing a persistent inflammatory milieu characteristic of inflammaging (Luo et al., 2024). In parallel, suppression of the nuclear factor erythroid 2–related factor 2 (Nrf2)–antioxidant response element (ARE) pathway diminishes transcription of endogenous antioxidant enzymes, including superoxide dismutase, catalase, and glutathione peroxidase, thereby exacerbating oxidative damage (Wang et al., 2022a). The reciprocal amplification between redox imbalance and inflammatory activation establishes a self-sustaining cycle that accelerates cellular dysfunction.

Mitochondria occupy a central position within this network. As primary sources and targets of ROS, dysfunctional mitochondria amplify oxidative stress, impair ATP production, and activate apoptotic signaling pathways (Zong et al., 2024). Accumulation of mitochondrial damage further exacerbates inflammatory signaling and promotes the emergence of senescence markers such as p16 and p21 (Zong et al., 2024; Fafián-Labora and O'Loghlen, 2020). Regulation of mitochondrial quality control is tightly governed by nutrient- and energy-sensing pathways, particularly the AMP-activated protein kinase (AMPK)–sirtuin-1 (SIRT1) axis and the mammalian target of rapamycin (mTOR) pathway (Watroba and Szukiewicz, 2021). In aging tissues, suppression of AMPK and SIRT1, together with chronic activation of mTOR signaling (Garcia and Shaw, 2017), impairs autophagic flux and mitochondrial turnover, thereby reinforcing redox and inflammatory dysregulation (Yu et al., 2021).

Importantly, metabolic and vascular aging are biologically interdependent processes linked by this redox–inflammatory–mitochondrial axis. Endothelial dysfunction, characterized by reduced nitric oxide (NO) bioavailability (Li et al., 2025b), increased oxidative burden, and heightened inflammatory signaling, directly contributes to impaired tissue perfusion and organ decline (Akhiyat et al., 2025). Uncoupling of endothelial nitric oxide synthase (eNOS) increases superoxide production, further activating NF-κB–mediated inflammation and perpetuating mitochondrial injury (Janaszak-Jasiecka et al., 2023). Thus, metabolic and vascular deterioration reinforce one another through shared molecular nodes, including AMPK–mTOR–SIRT1 signaling, Nrf2-mediated antioxidant defense, and NF-κB–driven inflammatory pathways.

Despite this mechanistic interdependence, pharmacological studies have largely examined metabolic and vascular aging as distinct entities. This compartmentalized perspective limits understanding of how coordinated modulation of shared redox and inflammatory pathways may provide a mechanistically plausible approach to attenuating age-associated decline. Therapeutic strategies targeting isolated components of this network may therefore underestimate the potential benefits of integrated pathway modulation.

Originally developed for glycemic control, empagliflozin, a sodium–glucose cotransporter-2 (SGLT2) inhibitor (Forycka et al., 2022), is now recognized to exert broad metabolic effects by modulating AMPK–SIRT1–mTOR signaling and rebalancing redox and inflammatory pathways (Forycka et al., 2022; Hasan et al., 2024).

Complementing these metabolic actions, nebivolol, a third-generation β_1_-selective adrenergic blocker with nitric oxide–modulating properties, targets the vascular arm of this axis by enhancing endothelial function, activating Nrf2-dependent antioxidant defenses, and suppressing NF-κB–mediated inflammatory signaling (Sabra et al., 2024). Although these agents originate from distinct pharmacological classes, they converge on key molecular pathways central to oxidative stress, inflammation, and mitochondrial regulation.

The present review synthesizes experimental and translational evidence to examine the effects of empagliflozin and nebivolol within an integrated metabolic–vascular aging framework. By focusing on the coordinated modulation of AMPK–mTOR–SIRT1, Nrf2, and NF-κB signaling, we aim to provide a mechanistic foundation for dual-pathway pharmacological strategies targeting the redox–inflammatory–mitochondrial axis underlying age-associated metabolic and vascular dysfunction.

## 2 Evidence hierarchy and critical appraisal

Despite growing interest in the potential aging-related effects of empagliflozin and nebivolol, the overall evidence base remains limited by substantial heterogeneity in study design, model selection, and translational relevance. Much of the currently available literature is derived from experimental disease-based models, particularly diabetes, cardiovascular dysfunction, hypertension, and oxidative injury paradigms, rather than physiological aging models. Consequently, several mechanistic interpretations of their proposed aging-relevant properties should be approached with caution. While both agents consistently demonstrate antioxidant, anti-inflammatory, and mitochondrial-modulating effects across multiple preclinical settings, these findings do not necessarily establish direct anti-aging efficacy.

Importantly, the strength of evidence differs considerably across mechanistic domains. Empagliflozin has comparatively stronger experimental and translational support, owing to a broader body of preclinical and clinical cardiovascular-metabolic research. However, many studies primarily evaluate secondary improvements in metabolic homeostasis, cardiac remodeling, or renal protection rather than aging-specific endpoints such as senescence burden, lifespan extension, or systemic functional decline. Similarly, nebivolol’s proposed aging-relevant role is largely extrapolated from endothelial and vascular studies demonstrating improvements in nitric oxide bioavailability, reductions in oxidative stress, and suppression of inflammation, with relatively limited direct investigation in established aging models.

In addition, the majority of cited mechanistic evidence comes from reductionist experimental approaches that focus on isolated molecular pathways or biochemical endpoints. Although modulation of AMPK–mTOR–SIRT1 signaling, Nrf2 activation, and NF-κB suppression is biologically relevant to aging, pathway convergence alone should not be interpreted as definitive evidence of coordinated therapeutic benefit (Kennedy et al., 2014). The complexity of aging biology introduces the possibility of compensatory signaling responses, tissue-specific variability, pharmacodynamic limitations, and context-dependent effects that remain insufficiently characterized.

Notably, no direct experimental or clinical studies have evaluated the combined administration of empagliflozin and nebivolol in the context of aging. Therefore, suggestions regarding additive or complementary interactions remain hypothetical and mechanistically inferred rather than empirically established. Future investigations should prioritize integrated, aging-specific models, multidimensional molecular profiling, and carefully controlled translational studies to distinguish mechanistic plausibility from validated, aging-relevant efficacy.

## 3 D-galactose-induced aging model: experimental representation of aging axis

The D-galactose–induced aging model is widely employed as a reproducible experimental paradigm to investigate the molecular mechanisms underlying accelerated senescence (Wang et al., 2022b). Chronic exposure to supraphysiological levels of D-galactose promotes abnormal oxidative metabolism and non-enzymatic glycation of macromolecules, leading to the formation of advanced glycation end products (AGEs) (Azman and Zakaria, 2019). The interaction of AGEs with their receptor (RAGE) initiates intracellular signaling cascades that activate NADPH oxidase and enhance ROS production, thereby reproducing key oxidative features observed in physiological aging (Lin et al., 2025). Elevated ROS levels disrupt redox homeostasis and trigger NF-κB activation, leading to increased transcription of pro-inflammatory mediators such as TNF-α, IL-6, and cyclo-oxygenase-2 (COX-2) (Yu et al., 2022). Concurrently, D-galactose exposure suppresses the Nrf2-ARE pathway, leading to reduced expression of endogenous antioxidant enzymes, including superoxide dismutase, catalase, and glutathione peroxidase (Ma et al., 2024). The combined activation of NF-κB and inhibition of Nrf2 establishes a self-perpetuating oxidative–inflammatory cycle that closely mirrors the molecular landscape of inflammaging. Mitochondrial dysfunction represents a central feature of this model. Excessive ROS accumulation impairs mitochondrial membrane integrity, reduces ATP production, and promotes apoptotic signaling (Guo et al., 2020).

These alterations intersect with nutrient-sensing pathways critical for cellular homeostasis. Suppression of AMPK and persistent activation of mTOR signaling have been consistently reported in D-galactose–treated tissues (Garcia and Shaw, 2017), contributing to impaired autophagic flux and the accumulation of damaged organelles (Li et al., 2025c). Reduced AMPK signaling further attenuates SIRT1 activity, diminishing stress adaptation and facilitating the accumulation of senescence-associated markers such as p16 and p21 (Li et al., 2025a).

At the tissue level, prolonged D-galactose administration induces structural and functional changes consistent with premature systemic aging, including neurodegeneration, myocardial remodeling, hepatocellular alterations, and endothelial thickening (Cai et al., 2022). These phenotypes reflect the integrated consequences of oxidative stress, inflammatory activation, mitochondrial dysfunction, and impaired cellular quality control.

**FIGURE 1:**
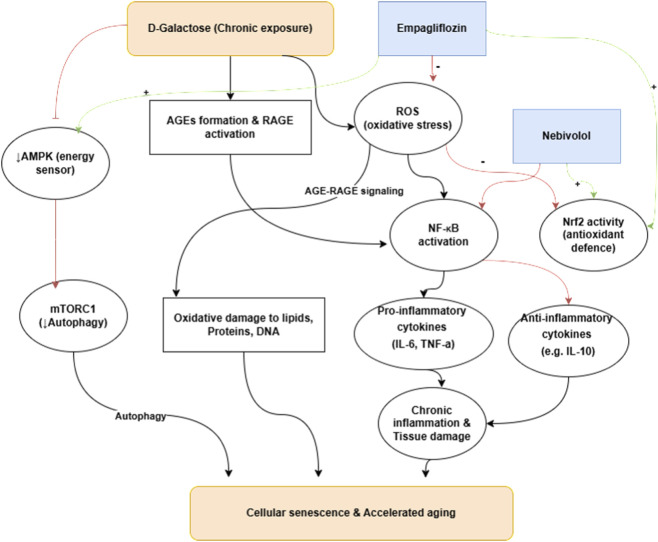
Proposed mechanistic crosstalk between D-galactose-induced oxidative stress and the dual modulatory actions of empagliflozin and nebivolol on aging-related molecular pathways. Chronic D-galactose exposure suppresses AMPK and activates mTORC1, leading to reduced autophagy, AGE–RAGE signaling, NF-κB activation, and oxidative damage. Empagliflozin and nebivolol counteract these effects by activating AMPK, enhancing Nrf2-mediated antioxidant defense, and inhibiting NF-κB–driven inflammation, thereby restoring redox balance and attenuating cellular senescence. Source: Created by the author based on the reviewed literature.

Although the D-galactose model represents an accelerated aging framework driven primarily by oxidative and glycation-related mechanisms, it does not fully recapitulate all aspects of chronological aging (Sadigh-Eteghad et al., 2017). Nevertheless, it remains a practical and mechanistically defined system for evaluating pharmacological interventions targeting redox imbalance and inflammaging. As summarized in Figure 1, D-galactose exposure promotes interconnected activation of AGE–RAGE/NF-κB signaling while suppressing adaptive AMPK- and Nrf2-mediated protective responses, thereby reinforcing oxidative-inflammatory and mitochondrial dysfunction associated with aging.

The model is characterized by well-established molecular endpoints, including malondialdehyde accumulation, depletion of antioxidant enzymes, NF-κB activation, and AMPK–mTOR dysregulation. These measurable markers permit quantitative assessment of agents that modulate the oxidative-inflammatory-mitochondrial axis. Accordingly, the D-galactose model provides a relevant platform for investigating pharmacological agents such as empagliflozin and nebivolol, whose pleiotropic actions converge on AMPK–mTOR-SIRT1 signaling, Nrf2-mediated antioxidant defense, and NF-κB–driven inflammatory pathways central to aging-related redox dysregulation.

## 4 Empagliflozin modulation of the metabolic–redox axis

Empagliflozin is widely used in the management of type 2 diabetes mellitus (Stevanovic et al., 2025). Beyond glycemic control, accumulating preclinical and clinical evidence indicates that it exerts pleiotropic effects on cellular pathways implicated in aging (Yesilyurt-Dirican et al., 2025; Obaid and Fawzi, 2024). These actions include modulation of nutrient-sensing signaling, attenuation of oxidative stress, suppression of inflammatory activation, and preservation of mitochondrial function. Its relevance to aging biology lies in its capacity to recalibrate energy-sensing networks and restore redox and inflammatory homeostasis, thereby counteracting molecular processes associated with senescence.

### 4.1 Regulation of AMPK–SIRT1–mTOR axis

Empagliflozin has been reported to modulate AMPK and mTOR signaling (Aman et al., 2021; Sa-Nguanmoo et al., 2017), thereby restoring autophagic flux and improving cellular quality-control mechanisms. Figure 1 illustrates how empagliflozin may counteract the metabolic component of the aging axis by activating AMPK, suppressing mTOR signaling, and restoring mitochondrial and autophagic homeostasis. AMPK activation also enhances SIRT1 expression, thereby supporting mitochondrial biogenesis, metabolic flexibility, and resilience to cellular stress. Through coordinated modulation of this axis, empagliflozin addresses core nutrient-sensing disturbances observed in aging tissues.

### 4.2 Modulation of oxidative stress via Nrf2 activation

Suppression of the Nrf2 pathway is a hallmark of aging-associated redox imbalance (Ngo and Duennwald, 2022). Empagliflozin has been reported to be associated with an enhanced Nrf2-mediated antioxidant signaling and with upregulation of downstream antioxidant enzymes, including superoxide dismutase, catalase, and glutathione peroxidase (Ozbek et al., 2025). This restoration of antioxidant defense reduces the accumulation of reactive oxygen species and mitigates oxidative damage. As depicted in Figure 1, reinforcement of Nrf2-dependent antioxidant signaling may interrupt the self-perpetuating oxidative-inflammatory cycle associated with D-galactose-induced aging. By stabilizing redox homeostasis, empagliflozin interrupts the oxidative component of the self-perpetuating redox–inflammatory cycle.

### 4.3 Inhibition of NF-κB–mediated inflammation

Chronic activation of NF-κB drives sustained production of pro-inflammatory mediators and contributes to the development of inflammaging (Mao et al., 2025; Sivandzade et al., 2019). Empagliflozin has been reported to suppress NF-κB activation and reduce transcription of inflammatory cytokines, including TNF-α and IL-6. By attenuating inflammatory signaling (Singh et al., 2021). Figure 1 further highlights the relationship among NF-κB activation, inflammatory amplification, and downstream mitochondrial dysfunction within the aging-associated redox-inflammatory network. Empagliflozin mitigates one of the principal drivers of age-associated metabolic and vascular dysfunction.

### 4.4 Mitochondrial function and cellular longevity

Coordinated regulation of AMPK–mTOR, Nrf2, and NF-κB signaling may contribute to preservation of mitochondrial function through interconnected effects on oxidative stress, inflammatory signaling, and cellular energy homeostasis (Rodríguez et al., 2021). Empagliflozin has been associated with the preservation of mitochondrial membrane potential, improved ATP production, and reduced apoptotic signaling (Cai et al., 2024; Sun et al., 2016). These effects may influence pathways associated with cellular senescence. By stabilizing mitochondrial integrity and reducing redox burden, empagliflozin supports cellular longevity pathways implicated in aging.

### 4.5 Systemic and translational implications

Beyond cellular mechanisms, empagliflozin confers systemic benefits relevant to aging phenotypes. Evidence from preclinical and clinical studies demonstrates improvements in metabolic homeostasis, endothelial function, and organ-level redox balance. However, most translational data derive from diabetic or cardiometabolic disease contexts, where disease-specific factors may confound interpretation of aging-related effects. Although empagliflozin exhibits mechanistic features associated with aging-related pathways. Together, these complementary mechanisms provide a mechanistically plausible framework for coordinated modulation of aging-associated pathways that requires further experimental validation. Importantly, much of the currently available evidence is derived from diabetes, heart failure, and broader cardiometabolic disease contexts rather than physiological aging models. Although these findings support potential mechanistic relevance to aging biology, direct evidence evaluating lifespan extension, health span improvement, frailty reduction, or non-diseased aging outcomes remains comparatively limited. Dedicated aging-focused experimental and clinical investigations are required to determine its efficacy, optimal dosing, and long-term safety in non-diseased aging populations, though recent *in-vivo* studies evaluating empagliflozin specifically as an anti-aging treatment have begun to address this gap (Obaid and Fawzi, 2024).

## 5 Nebivolol: targeting the vascular–redox axis

In contrast to the predominantly metabolic actions of empagliflozin, nebivolol exerts its pleiotropic effects primarily through endothelial and vascular mechanisms (Ginghina et al., 2026). Building upon the shared redox-inflammatory-mitochondrial framework, nebivolol targets nitric oxide bioavailability, endothelial oxidative balance, and inflammatory signaling (Fratta Pasini et al., 2005). These processes are central to vascular aging and systemic functional decline.

### 5.1 Nebivolol and eNOS–NO signaling

The eNOS uncoupling and reduced NO bioavailability are hallmarks of vascular aging (Janaszak-Jasiecka et al., 2023; Förstermann and Münzel, 2006). Impaired NO signaling promotes vasoconstriction, oxidative stress, and inflammatory activation (Bueno-Pereira et al., 2022). Nebivolol may uniquely improve endothelial function by stimulating eNOS activity and enhancing NO bioavailability (Garbin et al., 2008). The vascular arm of Figure 1 illustrates nebivolol’s proposed role in restoring nitric oxide bioavailability and improving endothelial redox balance during aging-associated vascular dysfunction, thereby enhancing vascular tone, tissue perfusion, and redox homeostasis (Gul et al., 2025). Restoration of NO signaling also limits superoxide generation and interrupts downstream inflammatory cascades.

### 5.2 Redox regulation via Nrf2 activation

Beyond its vasodilatory properties, nebivolol may support endothelial antioxidant defenses through Nrf2-associated pathways (Nascimento et al., 2022; Garbin et al., 2008). As summarized in Figure 1, nebivolol-mediated enhancement of antioxidant signaling may contribute to the stabilization of vascular oxidative homeostasis and attenuation of endothelial injury. Upregulation of antioxidant enzymes mitigates the accumulation of vascular reactive oxygen species and reduces oxidative injury. This action complements metabolic antioxidant mechanisms and reinforces redox equilibrium within aging vascular tissues.

### 5.3 Inhibition of NF-κB–mediated inflammation

Chronic activation of NF-κB contributes to endothelial dysfunction and vascular inflammaging (Pacinella et al., 2022; Sivandzade et al., 2019). Nebivolol suppresses NF-κB signaling and reduces the expression of pro-inflammatory mediators, thereby attenuating endothelial inflammation and improving vascular homeostasis (Sabra et al., 2024). Figure 1 further illustrates the interactions among vascular oxidative stress, NF-κB activation, and the inflammatory amplification targeted by nebivolol. By attenuating inflammatory signaling, nebivolol may directly attenuate the vascular component of the redox–inflammatory cycle that drives age-associated decline.

### 5.4 Mitochondrial integrity and endothelial senescence

Through coordinated improvements in NO signaling, redox balance, and inflammatory suppression, nebivolol may help preserve mitochondrial integrity in vascular tissues (Tran et al., 2022; Mason et al., 2005). Stabilization of mitochondrial function can reduce oxidative amplification and attenuate apoptotic signaling, thereby supporting endothelial homeostasis and vascular resilience during aging.

### 5.5 Translational considerations

At the systemic level, nebivolol has demonstrated beneficial effects on endothelial function, arterial stiffness, and vascular reactivity, parameters that are closely linked to age-related cardiovascular decline (Toblli et al., 2012). However, most translational evidence derives from hypertensive or cardiovascular disease populations, limiting definitive conclusions regarding aging-specific outcomes. Although nebivolol exhibits mechanistic features consistent with vascular aging-relevant potential, its role in healthy aging and its integration with metabolic-targeted interventions remain to be systematically investigated.

Furthermore, most available evidence regarding nebivolol is derived from vascular and cardiovascular research rather than dedicated aging-relevant investigations (Toblli et al., 2012). Consequently, its proposed relevance to aging remains largely mechanistically inferred, and direct studies evaluating lifespan, health span, or systemic aging-related outcomes remain scarce. Together, the metabolic-centric actions of empagliflozin and the vascular-focused mechanisms of nebivolol provide a compelling rationale for exploring coordinated modulation of shared molecular pathways in aging.

## 6 Mechanistic convergence and potential synergy

Metabolic and vascular dysfunction form a self-reinforcing cycle during aging, in which oxidative stress, inflammatory activation, and mitochondrial impairment amplify one another (Kennedy et al., 2014). The integrated metabolic–vascular framework summarized in Figure 1 highlights the mechanistic convergence of empagliflozin and nebivolol on interconnected aging-associated pathways involving AMPK–mTOR signaling, Nrf2-mediated antioxidant defense, NF-κB activation, mitochondrial dysfunction, and endothelial redox regulation.

Although empagliflozin and nebivolol belong to distinct pharmacological classes, they converge on shared molecular pathways that regulate redox balance, nutrient sensing, and cellular resilience. Empagliflozin predominantly targets metabolic reprogramming and mitochondrial quality control, whereas nebivolol acts primarily through endothelial nitric oxide signaling and vascular redox stabilization. Together, these complementary mechanisms provide a mechanistically plausible framework for the coordinated modulation of aging-associated pathways (Kennedy et al., 2014), which requires further experimental validation.

### 6.1 Convergent regulation of AMPK–mTOR–SIRT1 signaling

Dysregulation of the AMPK–SIRT1–mTOR axis contributes to impaired autophagy, mitochondrial dysfunction, and cellular senescence during aging (Ge et al., 2022). Emerging evidence suggests that empagliflozin modulates the AMPK–mTOR axis, potentially contributing to improved cellular energy homeostasis and enhanced autophagic activity (Ren et al., 2021). Nebivolol, by enhancing nitric oxide bioavailability and endothelial redox balance, indirectly influences AMPK–SIRT1 activity (Gul et al., 2022). These complementary actions suggest that combined metabolic and vascular modulation may support broader modulation of nutrient-sensing pathways than either intervention alone, although direct comparative studies remain unavailable.

### 6.2 Coordinated redox regulation via Nrf2 activation

Age-associated decline in Nrf2 signaling compromises endogenous antioxidant defenses and promotes oxidative injury. Evidence suggests that empagliflozin may enhance Nrf2-mediated antioxidant signaling by improving metabolic and mitochondrial homeostasis (Luna-Marco et al., 2024), whereas nebivolol appears to support endothelial antioxidant defenses via redox-sensitive antioxidant pathways (Petsouki et al., 2025). Together, these effects may reinforce redox homeostasis across intracellular and vascular compartments. Coordinated activation of antioxidant pathways may therefore interrupt the oxidative amplification that sustains inflammaging.

To contextualize the proposed metabolic–vascular framework, empagliflozin and nebivolol should be considered relative to established and emerging aging-relevant candidates. Compared with agents such as metformin, rapamycin, resveratrol, GLP-1 receptor agonists, NAD^+^ modulators, and senolytics, the aging-related evidence supporting empagliflozin and nebivolol remains comparatively limited and is derived predominantly from cardiometabolic and vascular disease models. Their relevance to aging should therefore be interpreted as mechanistically plausible rather than equivalent to established aging-relevant interventions. A comparative overview of established and emerging aging-relevant candidates is summarized in Table 1.

**TABLE 1:** Comparative positioning of empagliflozin and nebivolol relative to established aging-relevant candidates in aging-related research.

Agent/Class	Principal aging-related mechanisms	Evidence strength	Major limitations	Relevance to empagliflozin and nebivolol
Metformin	AMPK activation, mTOR suppression, improved insulin sensitivity, and anti-inflammatory effects	Strong preclinical evidence with growing translational aging research	Human aging-specific evidence remains limited; mechanisms may vary across tissues	Shares metabolic and AMPK-centered mechanisms, particularly with empagliflozin
Rapamycin	Direct mTORC1 inhibition, autophagy enhancement, nutrient-sensing modulation	Among the strongest preclinical longevity evidence	Immunosuppressive effects, metabolic adverse events, and translational safety concerns	Represents a more established mTOR-targeted aging-related strategy
Resveratrol	SIRT1 activation, AMPK modulation, antioxidant signaling	Moderate preclinical evidence	Poor bioavailability and inconsistent clinical translation	Mechanistically overlaps with AMPK–SIRT1 and Nrf2-related pathways
GLP-1 receptor agonist	Metabolic regulation, anti-inflammatory effects, cardiovascular protection	Increasing clinical and translational evidence	Aging-specific evidence remains preliminary	Mechanistically overlaps with AMPK–SIRT1 and Nrf2-related pathways
NAD+ modulators	Mitochondrial function, DNA repair support, sirtuin activation	Promising experimental evidence with early clinical investigation	Long-term efficacy and optimal dosing remain uncertain	Overlaps with mitochondrial and SIRT1-associated pathways
Senolytics	Elimination of senescent cells, reduction of senescence-associated secretory phenotype (SASP)	Strong experimental interest in emerging clinical trials	Tissue specificity, safety, and dosing strategies remain unresolved	More directly targets cellular senescence than empagliflozin or nebivolol
Empagliflozin	AMPK–mTOR modulation, Nrf2 activation, NF-κB suppression, mitochondrial protection	Moderate experimental and cardiometabolic evidence; limited direct aging evidence	Predominantly disease-model-based evidence	Primarily targets metabolic-redox components of aging pathways
Nebivolol	Nitric oxide bioavailability, endothelial protection, Nrf2 activation, NF-κB suppression	Moderate vascular and endothelial evidence; limited aging-specific research	Evidence largely extrapolated from cardiovascular studies	Primarily targets vascular-redox and inflammatory components of aging pathways

In addition to broader comparison with established aging-relevant candidates, Table 2 summarizes the currently available mechanistic evidence supporting empagliflozin and nebivolol in aging-associated molecular pathways.

**TABLE 2:** Evidence supporting the effects of empagliflozin and nebivolol on aging-related pathways.

Agent	Aging-related pathway/process	Reported mechanistic effect	Evidence source/type	Critical limitation
Empagliflozin	AMPK–mTOR–SIRT1 axis	Associated with the modulation of AMPK signaling, suppresses mTOR signaling, and supports autophagy-related cellular quality control	Mainly preclinical cardiometabolic and disease-based models	Limited direct evidence in physiological aging models
Empagliflozin	Oxidative stress/Nrf2 signaling	Associated with antioxidant pathway modulation through Nrf2-related pathways and reduces ROS-associated injury	Experimental metabolic, renal, and cardiovascular models	Findings are often organ- or disease-specific
Empagliflozin	NF-κB-mediated inflammation	Reduces inflammatory signaling and pro-inflammatory cytokine expression	Preclinical and translational cardiometabolic studies	Anti-inflammatory effects are not consistently validated as aging-specific
Empagliflozin	Mitochondrial function	Improves mitochondrial integrity, energy balance, and resistance to oxidative injury	Mostly experimental disease models	Lifespan, frailty, and senescence outcomes remain insufficiently studied
Nebivolol	Endothelial NO/eNOS signaling	Enhances nitric oxide bioavailability and improves endothelial function	Vascular and cardiovascular studies	Evidence is mainly extrapolated from hypertension and cardiovascular disease
Nebivolol	Oxidative stress/Nrf2 signaling	Reduces vascular oxidative stress and may enhance antioxidant responses	Endothelial and cardiovascular experimental models	Direct validation in aging models is limited
Nebivolol	NF-κB-mediated inflammation	Suppresses inflammatory activation and reduces vascular inflammatory tone	Preclinical vascular and cardiovascular evidence	Aging-specific inflammatory endpoints remain underexplored
Nebivolol	Mitochondrial/endothelial senescence	May preserve mitochondrial integrity indirectly through improved redox and NO signaling	Mostly mechanistic and endothelial studies	Effects on cellular senescence are largely inferred rather than directly proven
Empagliflozin + nebivolol	Coordinated metabolic–vascular modulation	Mechanistically plausible convergence on AMPK–mTOR, Nrf2, NF-κB, mitochondrial function, and endothelial homeostasis	No direct combination studies in aging are currently available	​

## 7 Pharmacodynamic uncertainty and potential limitations

Despite the mechanistic convergence described above, coordinated modulation of aging-associated pathways does not necessarily translate into definitive therapeutic synergy. Aging biology involves complex and context-dependent interactions among metabolic, inflammatory, mitochondrial, and vascular signaling networks. Consequently, pharmacological interventions targeting shared pathways may produce variable or tissue-specific effects depending on baseline metabolic status, disease context, treatment duration, and compensatory signaling responses.

In addition, not all downstream consequences of AMPK activation, mTOR suppression, or inflammatory modulation are uniformly beneficial across physiological settings. Chronic pathway modulation may induce adaptive metabolic alterations, excessive suppression of nutrient sensing, or unintended effects on cellular stress responses. Furthermore, the absence of direct combination studies evaluating empagliflozin and nebivolol introduces significant uncertainty regarding pharmacodynamic interactions, optimal dosing strategies, and long-term safety.

Therefore, although the available evidence supports a biologically plausible framework for coordinated metabolic–vascular modulation, mechanistic convergence alone should not be interpreted as definitive evidence of additive or synergistic geroprotective efficacy. Further aging-specific experimental and translational investigations are required to clarify potential therapeutic benefits, limitations, and context-dependent responses associated with combined pathway modulation.

## 8 Knowledge gaps and future directions

Despite the integrated metabolic–vascular framework outlined above, several important gaps remain in the pharmacological targeting of aging. Although empagliflozin and nebivolol individually modulate key longevity-associated pathways, their combined effects within an aging context have not been systematically investigated. To date, no studies have evaluated concurrent administration of these agents to determine whether their complementary mechanisms yield additive, complementary, or context-dependent benefits on oxidative stress, inflammation, mitochondrial dysfunction, and cellular senescence.

A further limitation lies in the predominance of reductionist experimental approaches. Much of the existing literature relies on isolated biochemical or histological endpoints, which do not fully capture the complexity of pathway crosstalk that drives aging. Systems-level methodologies, including transcriptomics, proteomics, metabolomics, and phosphoproteomics, are needed to delineate temporal and hierarchical interactions among AMPK–SIRT1–mTOR signaling, NF-κB activation, Nrf2-mediated antioxidant responses, and mitochondrial networks. Such integrative strategies would provide deeper insight into coordinated metabolic–vascular regulation during pharmacological intervention.

Translational evidence remains limited; most clinical data for empagliflozin and nebivolol derive from populations with diabetes, hypertension, or cardiovascular disease. While informative, these disease-specific contexts may confound the interpretation of aging-related mechanisms. The implications of these findings for physiological aging in non-diseased individuals are therefore uncertain. Critical questions remain unresolved, including optimal dosing strategies, treatment duration, long-term safety, and potential drug–drug interactions in older adults.

Future research should prioritize integrated experimental models that combine aging-specific animal systems with multidimensional molecular profiling and carefully designed translational studies. Such approaches may clarify whether coordinated metabolic–vascular modulation represents a viable strategy to delay functional decline and reduce the burden of age-related disease. Addressing these gaps will be essential to advance from pathway-specific interventions toward a systems-level aging-relevant paradigm.

## 9 Conclusion

Aging is driven by an interconnected network of oxidative stress, chronic inflammation, mitochondrial dysfunction, and nutrient-sensing imbalance. A potential intervention strategy, therefore, requires modulation of multiple, interdependent molecular pathways rather than isolated targets. Empagliflozin and nebivolol, although derived from distinct pharmacological classes, converge mechanistically on key longevity-associated signaling nodes, including AMPK–mTOR–SIRT1, Nrf2, and NF-κB. These pathways collectively regulate cellular energy homeostasis, redox balance, autophagy, and inflammatory tone. Empagliflozin has been reported to influence metabolic signaling pathways, including AMPK–SIRT1, inhibit mTOR signaling, and enhance Nrf2-dependent antioxidant defenses, thereby improving mitochondrial integrity and suppressing inflammatory activation. Nebivolol complements these actions by restoring endothelial nitric oxide bioavailability, attenuating NF-κB–mediated inflammation, and reinforcing vascular redox stability. Together, these mechanisms illustrate a plausible framework for coordinated metabolic–vascular modulation of aging-related pathways. Although direct experimental and translational evidence supporting combined administration remains limited, the proposed complementary interaction between empagliflozin and nebivolol should currently be interpreted as mechanistically plausible rather than experimentally established. The shared targeting of redox–inflammatory–mitochondrial networks provides a strong mechanistic rationale for further investigation. Systematic studies, including aging-focused experimental models and carefully designed clinical research, are necessary to determine whether integrated metabolic and vascular modulation can meaningfully attenuate functional decline and reduce the burden of age-associated disease. Advancing from pathway-specific observations toward a systems-level aging-relevant.

The strategy will require convergence of mechanistic insight and translational validation. Importantly, mechanistic convergence between pharmacological agents does not necessarily imply therapeutic complementarity, as pathway interactions may be context-dependent and influenced by dose, tissue specificity, and disease state.
